# Clinical Outcomes and Healthcare Utilization in Pulmonary Embolism Patients With and Without Prothrombin G20210A Mutation: A National Retrospective Cohort Study

**DOI:** 10.7759/cureus.76921

**Published:** 2025-01-04

**Authors:** Fahmida Sultana, Ribaba Rayan, Tasnim Jabbar Brishti, Iffath Mizan, Sabrina Sifat, FNU Ifatujjahan, Juan Carlos Sequeira Gross, Garry Aliosha Francis Morel

**Affiliations:** 1 Medicine, Washington University of Health and Sciences, San Pedro, BLZ; 2 Internal Medicine, Shaheed Suhrawardy Medical College and Hospital, Dhaka, BGD; 3 Medicine, Ibrahim Medical College, Dhaka, BGD; 4 Medicine, El Camino Hospital, Mountain View, USA; 5 Medicine, Kumudini Women's Medical College, Tangail, BGD; 6 Medicine, Shaheed Ziaur Rahman Medical College, Bogra, BGD; 7 Internal Medicine, Lee Memorial Health System, Gulf Coast Medical Center, Fort Myers, USA; 8 Department of Medicine, Division of Pulmonary, Critical Care and Sleep Medicine, Saint Louis University Hospital, St. Louis, USA

**Keywords:** comorbidities, g20210a, mortality, national inpatient sample, prothrombin gene mutation, pulmonary embolism

## Abstract

Background: Pulmonary embolism (PE) is a severe condition often linked to thromboembolic risk factors, such as the prothrombin gene (PGM) G20210A mutation. Although this mutation is a recognized risk factor for venous thromboembolism, little is known about how it affects the clinical course and healthcare utilization of PE patients.

Objective: This new study will reveal the complete effect of PGM on clinical outcomes in patients treated for PE, such as hospital stay length, in-hospital death rates, healthcare costs, and associated health conditions. It will also examine how socioeconomic status and demographics impact these outcomes.

Methods: This retrospective cohort study was conducted using data from the National Inpatient Sample (NIS) from 2016 to 2020. It included adults admitted with a primary diagnosis of PE aged 18 and older. The patients were divided into two groups: those with the PGM mutation and those without. There were two groups where the patients was divided based on their PGM use: those with and those without. Multivariate logistic regression assessed in-hospital mortality, while linear regression models evaluated length of stay (LOS) and healthcare charges. The models were adjusted for demographics, comorbidities (Charlson Comorbidity Index), and hospital characteristics.

Results: Among the 903,230 PE patients, 2,065 (0.2%) had PGM. Patients with the mutation had a significantly lower in-hospital mortality than those without the mutation (adjusted OR 0.13, 95% CI 0.02-0.92, p = 0.041). PGM carriers also had lower rates of atrial fibrillation (5.1% vs. 11.8%, p < 0.001), congestive heart failure (CHF) (5.9% vs. 16.0%, p < 0.001), and chronic obstructive pulmonary disease (COPD) (8.6% vs. 15.5%, p < 0.001), but higher rates of obesity (32.5% vs. 26.1%, p = 0.004) and hyperlipidemia (30.7% vs. 36.0%, p = 0.031). Despite a longer hospital stay in PGM patients (mean difference: 0.52 days, p = 0.005), the difference in total hospital charges was not statistically significant (mean difference: $6,295, p = 0.090).

Conclusions: Patients with PE and the PGM had lower mortality rates in this national retrospective cohort than those without the mutation. Patients without the PGM presented with more serious comorbidities, including higher rates of atrial fibrillation, CHF, and COPD, which may have contributed to their worse outcomes.

## Introduction

When a blood clot (pulmonary embolism (PE)) occurs, which is a potentially life-threatening condition, it usually commonly develops in the deep veins of the legs and pelvis, or occasionally the arms and travels to the lungs. Upon reaching the pulmonary arteries, the clot can obstruct blood flow, placing intense pressure on the lungs and heart. This blockage can have dangerous and sudden effects; if it is not treated immediately, it often leads to serious complications. Rapid diagnosis and timely medical intervention are essential to ensure the possible outcome and to prevent further damage [1]. Rapid diagnosis and timely medical intervention are essential to ensure the possible outcome and to prevent further damage. Every year, approximately 900,000 individuals in the United States face the serious threat of PE, a condition that leads to an estimated 60,000 to 100,000 fatalities. One of the most alarming aspects of PE is that for around 25% of those affected, the first indication of this life-threatening condition is sudden death. This stark reality underscores the urgent need for swift diagnosis and prompt treatment, as these factors can significantly enhance survival chances. It's a reminder of how critical it is for patients and healthcare providers to be vigilant about PE risks and act quickly when symptoms arise [1].

PE happens when multiple factors come into play, including both things we acquire over time and genetic predispositions. For instance, having surgery, being immobilized for a long period, or dealing with cancer can increase the risk of PE [2]. On the genetic side, one particular mutation in the prothrombin gene (PGM) has been studied extensively. This G20210A mutation increases prothrombin levels in the blood, making the formation of clots more likely and raising the risk of a pulmonary embolism [3]. Previous studies have shown that individuals with this mutation are more likely to develop deep vein thrombosis (DVT), which can result in PE [4].

Even though we recognize that the PGM mutation is connected to thromboembolic events, there aren’t many thorough studies that compare patients with PE who have this mutation to those who don’t. This gap is concerning because it limits our understanding of how this mutation might affect patient outcomes and treatment options [5]. In this area, if more research can be done, it can shed light on its significance [6]. Understanding how the PGM influences the prevalence of comorbidities, in-hospital mortality rates, and healthcare resource use is vital for developing targeted treatment approaches [7].

By analyzing the data, this study aims to bridge that gap from the National Inpatient Sample (NIS) to explore clinical outcomes and healthcare costs associated with PE in both groups. By comparing these two cohorts, we hope to uncover significant predictors of mortality, duration of hospital stays, and overall healthcare expenses. What we learn can influence healthcare policies, helping us improve how we practice medicine and achieve better patient outcomes. It’s all about making sure we provide the best care possible.

## Materials and methods

Data source

The protocols of Strengthening the Reporting of Observational Studies in Epidemiology (STROBE) were adhered to in this investigation [8] and conducted a retrospective analysis over five years (2016-2020). The data were sourced from the National Inpatient Sample (NIS), a key component of the Healthcare Cost and Utilization Project (HCUP) which is overseen by the Agency for Healthcare Research and Quality (AHRQ). HCUP, a collaborative effort involving federal, state, and industry partners, provides extensive healthcare databases and analytical tools. The NIS, recognized as the largest all-payer inpatient database in the United States, offers a representative sample of hospitalizations excluding long-term care and rehabilitation settings. Our analysis targeted patients diagnosed with PE, with and without PGM, over the defined study period. The data were classified using the International Classification of Diseases, Tenth Revision (ICD-10) and Procedure Coding System (ICD-10-PCS). The application of weighted data ensures that the results reflect approximately 97% of the U.S. population, improving the general applicability of the findings [9].

Study population

Using ICD-10 codes (Table 1), patients with PE as their major diagnosis were located (I26.92, I26.93, I26.94, I26.99, I26.02, I26.09). It was established that the secondary diagnostic code D68.52 indicated the existence of the PGM. Two cohorts were created from the study population: patients with PE and PGM, and those with PE without the mutation. Only non-elective admissions between January 2016 and December 2020 were included to ensure that acute cases were captured. Patients under the age of 18 were not included in the analysis.

**Table 1:** ICD-10-CM Diagnosis Code ICD-10-CM Code: International Classification of Diseases, 10th Revision, Clinical Modification Codes used to classify medical conditions. Condition: The specific medical condition associated with the listed ICD-10-CM codes. Description: A brief explanation of the listed condition.

ICD-10-CM Code	Condition	Description
"I480" "I481" "I482" "I4891"	Unspecified atrial fibrillation (Afib)	Atrial fibrillation without further specification
"I2583" "I2584" "I2510" "I25110" "I25111" "I25118" "I25119"	Atherosclerotic heart disease of native coronary artery without angina pectoris (CAD)	Coronary artery disease without symptoms of chest pain
"I5040" "I5041" "I5042" "I5043" "I509" "I5030" "I5031" "I5032" "I5033" "I5020" "I5021" "I5022" "I5023"	Congestive heart failure NOS, unspecified (CHF)	Heart failure without further specification
"I7389" "I739" "I739"	Peripheral vascular disease, unspecified (PVD)	Circulatory system disorders affecting blood vessels outside of the heart and brain
"I151" "I152" "I158" "I159" "I160" "I161" "I169"	Essential (primary) hypertension (HTN)	High blood pressure without secondary causes
E66.9	Obesity, unspecified	Excessive body weight without further specification
"E782" "E784" "E785" "E7800" "E7801" "E781"	Hyperlipidemia, unspecified (HLD)	Elevated cholesterol or fat levels in the blood without further specification
"Z8673" "I6300" "I63011" "I63012" "I63013" "I63019" "I6302" "I63031" "I63032" "I63033" "I63039" "I6309" "I6310" "I63111" "I63112" "I63113" "I63119" "I6312" "I63131" "I63132" "I63133" "I63139" "I6319" "I6320" "I63211" "I63212" "I63213" "I63219" "I6322" "I63231" "I63232" "I63233" "I63239" "I6329" "I6330" "I63311" "I63312" "I63313" "I63319" "I63321" "I63322" "I63323" "I63329" "I63331" "I63332" "I63333" "I63339" "I63341" "I63342" "I63343" "I63349" "I6339" "I6340" "I63411" "I63412" "I63413" "I63419" "I63421" "I63422" "I63423" "I63429" "I63431" "I63432" "I63433" "I63439" "I63441" "I63442" "I63443" "I63449" "I6349" "I6350" "I63511" "I63512" "I63513" "I63519" "I63521" "I63522" "I63523" "I63529" "I63531" "I63532" "I63533" "I63539" "I63541" "I63542" "I63543" "I63549" "I6359" "I636" "I638" "I639"	Cerebral infarction, unspecified (CVA)	Stroke due to a blockage or bleed in the brain without further specification
J44.9	Chronic obstructive pulmonary disease, unspecified (COPD)	Long-term lung disease causing breathing difficulties without further specification
N18.6	End stage renal disease (ESRD)	Advanced kidney failure requiring dialysis or transplantation
K74.60	Unspecified cirrhosis of liver (Cirrhosis)	Scarring of the liver without further specification
J80	Acute respiratory distress syndrome (ARDS)	Severe lung condition causing low oxygen levels in the blood
I21.9	Acute myocardial infarction, unspecified (MI)	Heart attack without further specification
F03	Unspecified dementia	Cognitive decline without a known cause
N17.9	Acute kidney failure, unspecified, applicable to Acute renal failure on dialysis (AKI_HD)	Sudden loss of kidney function, may require dialysis
Z99.11	Dependence on respirator [ventilator] status (MV)	Long-term dependence on a mechanical ventilator
Z86.711	History of pulmonary embolism on long-term anticoagulation therapy (Thrombolysis)	Previous pulmonary embolism with ongoing blood-thinning treatment

Study endpoints

The study's primary endpoint was to compare all-cause in-hospital mortality between two cohorts: hospitalizations for PE without concomitant PGM versus those with it. Secondary outcomes included comparisons of hospital length of stay (LOS) and total charges. Demographic data, patient characteristics, and comorbidities were also detailed.

Statistical analysis

All statistical analyses were performed using Stata MP/18 (StataCorp., College Station, TX, USA), and graphics were generated using RStudio software (Posit PBC, Boston, MA, USA). Descriptive statistics were used to compare the characteristics baseline between patients with and without PGM. Categorical variables were analyzed using the χ² test, and continuous variables were analyzed using the Student's t-test. Multivariable models were created to consider possible confounders to evaluate the association between the PGM status and key outcomes. Using multivariable logistic regression, we looked at in-hospital mortality to get adjusted odds ratios (ORs). In doing so, we considered various factors, including age, sex, race/ethnicity, household income quartiles, and important hospital characteristics like size and whether they teach hospitals. This comprehensive approach helped us better understand the influences on mortality. We have also used the Charlson Comorbidity Index (CCI) to assess the severity of illnesses among the patients. Multivariable linear regression identified predictors of LOS and total hospital charges using the same covariates. The survey weights from the NIS ensured nationally representative estimates. The survey design was specified using the svyset command, with adjustments for hospital clustering and stratification. All the analyses have accounted for the complex survey design through survey-weighted regression models. In addition to descriptive and regression analyses, subpopulation analyses assessed the outcomes among patients hospitalized for PE. The statistical significance was set at a two-tailed p-value of less than 0.05.

Patient confidentiality and institutional board review

In compliance with the Health Insurance Portability and Accountability Act (HIPAA) Privacy Rule under 45 CFR § 164.514(e), the NIS data is classified as a limited dataset. By removing 16 direct identifiers detailed in 45 CFR § 164.514(e)(2), limited datasets are exempt from Institutional Review Board (IRB) approval.

## Results

Demographics and comorbidities 

**Figure 1:**
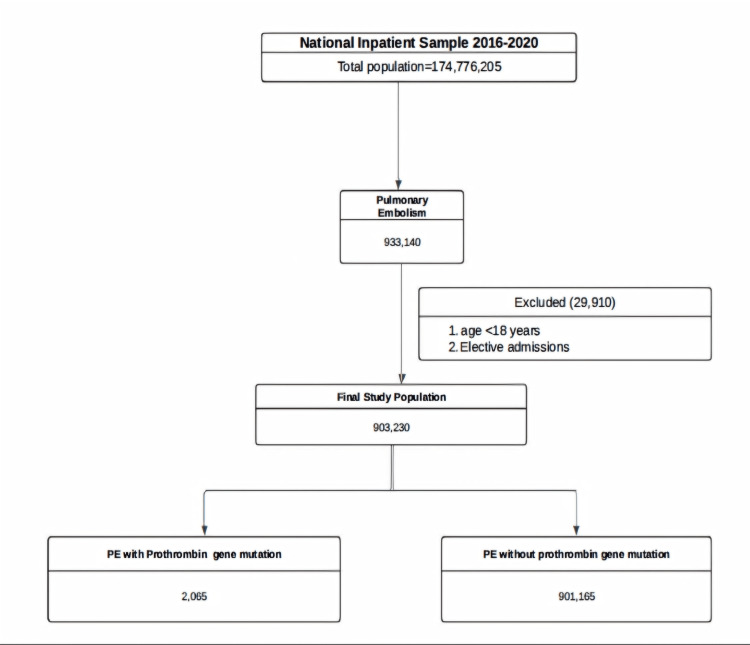
Flowchart PE: Pulmonary embolism

Among the 903,230 patients hospitalized for PE, 2,065 (0.2%) were identified as having the PGM G20210A (Figure 1). PGM patients were generally younger, with a mean age of 52.8 years compared to 63.0 years in non-PGM patients (Table 2). The distribution of sex was similar between the groups, with 53.9% of PGM patients being female, compared to 51.7% of non-PGM patients. A higher proportion of PGM patients were white (78.5% vs. 71.2%), whereas fewer were black (11.0% vs. 19.4%). PGM patients were more likely to be in higher income brackets, with 29.6% in the highest income quartile compared to 20.3% in non-PGM patients (Table 2). The comorbidity profiles between the two groups were notably different. The comorbidity profiles differed significantly between the two groups. The profiles of comorbidities showed notable differences between the two groups. Those with the PGM mutation had lower rates of conditions associated with poorer outcomes in pulmonary embolism, such as atrial fibrillation (5.1% vs. 11.8%), congestive heart failure (5.9% vs. 16.0%), and chronic obstructive pulmonary disease (8.6% vs. 15.5%). Conversely, obesity was more prevalent in PGM patients (32.5% vs. 26.1%) (Table 3).

**Table 2:** Demographic Characteristics of Patients Admitted with Pulmonary Embolism PE: Pulmonary embolism; PGM: Prothrombin gene mutation; p value <0.05: Statistical Significance; PI: Pacific Islander; N/A: Not applicable; N: Number of patients; LOS: Mean Length of Stay in the hospital in days; Total hospital charges: Mean total charges incurred during the hospital stay in US dollars.

Parameters	Total	PE with PGM (n = 2,065)	PE without PGM (n = 901,165)	P-value
Number of cases	903,230	0.2 %	99.8 %	N/A
Mean age (years)	63	52.8	63.0	<0.001
Sex (%)				
Male	48.2	46.1	48.3	0.384
Female	51.8	53.9	51.7	
Race (%)				
White	71.2	78.5	71.2	
Black	19.3	11.0	19.4	
Hispanic	6.0	7.4	5.8	<0.001
Asian or PI	1.0	0.2	1.0	
Native American	0.4	2.8	2.2	
Other	2.1	0.01	0.4	
Median household income (%)				
$1 - $49,999	28.5	21.4	28.5	
$50,000 - $64,999	26.5	21.5	26.5	<0.001
$65,000 - $85,999	24.7	27.5	24.7	
$86,000 or more	20.3	29.6	20.3	
Charlson Comorbidity Index (%)				
0	30.2	43.7	30.0	
1	23.3	30.5	23.4	<0.001
2	16.6	13.5	16.7	
3	29.9	12.3	29.9	
Insurance (%)				
Medicare	53.6	32.2	53.6	
Medicaid	12.4	17.5	12.4	<0.001
Private	29.7	46.5	29.7	
No insurance	4.3	3.8	4.3	
Region of Hospital (%)				
Northeast	18.3	22.1	18.3	
Midwest	25.0	29.0	25.0	0.014
South	38.7	31.0	38.7	
West	18.0	17.9	18.0	
Relative Bed Size (%)				
Small	21.3	19.7	21.3	
Medium	29.5	29.3	29.5	0.721
Large	49.2	51.0	49.2	
For-profit status (%)				
No	88.2	94.8	88.2	<0.001
Yes	11.8	5.2	11.8	
Teaching hospital status (%)				
No	30.8	25.8	30.8	0.037
Yes	69.2	74.2	69.2	
LOS	4.32	4.33	4.32	0.956
Hospital Charges	50,090	54,321	50,081	0.236
Died during hospitalization (%)	3.14	0.49	3.14	0.002

**Table 3:** Comorbidity Profile in Patients with Pulmonary Embolism PE: Pulmonary embolism; PGM: Prothrombin gene mutation; p value <0.05: statistical significance; MI: myocardial infarction; ARDS: acute respiratory distress syndrome; HLD: hyperlipidemia; PVD: peripheral vascular disease; CHF: congestive heart failure; CAD: coronary artery disease; Afib: atrial fibrillation, HTN: hypertension; CVA: cerebrovascular accidents; COPD: chronic obstructive pulmonary disease; ESRD: end stage renal disease; AKI-HD: Acute kidney injury requiring hemodialysis; MV: mechanical ventilation

Comorbidities (%)	PE with PGM (n = 2,065)	PE without PGM (n = 901,165)	P-value
Afib	5.1	11.8	<0.001
CAD	12.3	16.0	0.043
CHF	5.9	16.0	<0.001
PVD	1.4	2.1	0.377
HTN	37.4	42.2	0.060
Obesity	32.5	26.1	0.004
HLD	30.7	36.0	0.031
CVA	6.4	6.1	0.853
COPD	8.6	15.5	<0.001
ESRD	0.7	1.2	0.371
Cirrhosis	0.2	0.9	0.156
ARDS	0.2	0.1	0.507
MI	1.4	1.8	0.661
Dementia	0.7	5.7	<0.001
Vasopressors	0.4	0.8	0.450
AKI-HD	0	0.1	0.424
MV	1.4	3.3	0.037
Thrombolysis	4.1	3.1	0.204

In-hospital mortality

Patients with PGM mutations had a noticeably lower risk of death during their hospital stay compared to those without the mutation. The odds ratio for in-hospital mortality that was adjusted among PGM carriers was 0.13 (95% CI 0.02-0.92), meaning their chances of surviving were 87% higher than patients without the PGM mutation (as shown in Table 4).

**Table 4:** Predictors of Mortality aOR: adjusted Odds Ratio; p-value: significance defined as <0.05, PE: Pulmonary embolism; PGM: Prothrombin gene mutation; 95% Confidence Interval: A range that estimates where the true value of the parameter lies with 95% confidence, Ref: Reference group, NI: Not Included, $: Income range in U.S. dollars.

Parameters	aOR	95% confidence interval	P-value
PE with PGM	0.13	0.02 | 0.92	0.041
Age	1.02	1.02 | 1.02	<0.001
Sex			
Male	Ref	Ref	NI
Female	1.05	0.99 | 1.11	0.114
Race			
White	Ref	Ref	NI
Black	1.14	1.05 | 1.22	0.001
Hispanic	1.22	1.08 | 1.37	0.001
Asian or Pacific Islander	1.66	1.33 | 2.07	<0.001
Native American	1.35	0.85 | 2.14	0.205
Others	1.54	1.29 | 1.83	<0.001
Median Household Income			
$1 - $49,999	Ref	Ref	NI
$50,000 - $64,999	0.95	0.88 | 1.03	0.225
$65,000 - $85,999	0.92	0.85 | 0.99	0.032
$86,000 or more	0.91	0.84 | 1.00	0.038
Charlson index	1.19	1.18 | 1.21	<0.001
Insurance			
Medicare	Ref	Ref	NI
Medicaid	1.06	0.94 | 1.19	0.325
Private	1.04	0.96 | 1.13	0.357
No insurance	1.52	1.29 | 1.78	<0.001
Hospital Bed Size			
Small	Ref	Ref	NI
Medium	1.27	1.16 | 1.39	<0.001
Large	1.49	1.38 | 1.62	<0.001
Teaching status			
Non-teaching	Ref	Ref	NI
Teaching	1.45	1.36 | 1.55	<0.001

Length of hospital stay 

Patients with PGM mutations tended to stay in the hospital slightly longer than those without the mutation. After adjustments, the average length of stay was 0.52 days longer for PGM carriers (95% CI 0.16-0.88), as outlined in Table 5.

**Table 5:** Predictors of Length of stay LOS: Length of Stay, P-value: significance defined as <0.05, Coefficient: The estimated effect size of the variable on the outcome, 95% Confidence Interval: A range that estimates where the true value of the coefficient lies with 95% confidence, Ref: Reference group (used as the baseline comparison group in statistical analysis), NI: Not Included (indicates that the variable was not included in the statistical analysis), $: Income range in U.S. dollars, PE: Pulmonary Embolism, PGM: Prothrombin Gene Mutation.

Parameters	Coefficient	95% confidence interval	P-value
PE with PGM	0.52	0.16 | 0.88	0.005
Age	0.01	0.01 | 0.01	<0.001
Sex			
Male	Ref	Ref	NI
Female	0.15	0.10 | 0.19	<0.001
Race			
White	Ref	Ref	NI
Black	0.53	0.46 | 0.60	<0.001
Hispanic	0.21	0.12 | 0.31	<0.001
Asian or Pacific Islander	0.26	-0.01 | 0.52	0.055
Native American	0.02	-0.33 | 0.36	0.924
Others	0.54	0.32 | 0.76	<0.001
Median Household Income			
$1 - $49,999	Ref	Ref	NI
$50,000 - $64,999	-0.11	-0.17 | -0.05	0.001
$65,000 - $85,999	-0.16	-0.22 | -0.09	<0.001
$86,000 or more	-0.23	-0.30 | -0.16	<0.001
Charlson index	0.32	0.31 | 0.33	<0.001
Insurance			
Medicare	Ref	Ref	NI
Medicaid	0.32	0.21 | 0.42	<0.001
Private	-0.36	-0.43 | -0.30	<0.001
No insurance	0.25	0.12 | 0.39	<0.001
Hospital Bed Size			
Small	Ref	Ref	NI
Medium	0.40	0.34 | 0.47	<0.001
Large	0.75	0.69 | 0.81	<0.001
Teaching status			
Non-teaching	Ref	Ref	NI
Teaching	0.62	0.57 | 0.67	<0.001

Hospital charges

There was no significant difference in the total hospital charge between the PGM and non-PGM patients. The average difference in total hospital charges for patients was $6,295, but there was a lot of variation in these figures. In the wide confidence interval, this is reflected (95% CI: -$982 to $13,573) shown in Table 6, indicating that hospital costs can vary significantly from one patient to another.

**Table 6:** Predictors of Total Charges P-value: significance defined as <0.05, Coefficient: The estimated effect size of the variable on the outcome (in this case, likely a monetary value or LOS), 95% Confidence Interval: A range that estimates where the true value of the coefficient lies with 95% confidence, Ref: Reference group (used as the baseline comparison group in statistical analysis), NI: Not Included (indicates that the variable was not included in the statistical analysis), $: Income range in U.S. dollars, PE: Pulmonary Embolism, PGM: Prothrombin Gene Mutation, Charlson Index: A measure of comorbidity that considers the number and severity of chronic conditions in patients.

Parameters	Coefficient	95% confidence interval	P- value
PE with PGM	6295	-982 | 13573	0.090
Age	-28	-59 | 2	0.072
Sex			
Male	Ref	Ref	NI
Female	-758	-1434 | -83	0.028
Race			
White	Ref	Ref	NI
Black	5871	4666 | 7077	<0.001
Hispanic	16845	14827 | 18864	<0.001
Asian or Pacific Islander	17168	12225 | 22111	<0.001
Native American	-3900	-9058 | 1258	0.138
Others	14681	11168 | 18193	<0.001
Median Household Income			
$1 - $49,999	Ref	Ref	NI
$50,000 - $64,999	778	-247 | 1802	0.137
$65,000 - $85,999	1757	622 | 2893	0.002
$86,000 or more	5709	4281 | 7137	<0.001
Charlson index	3156	2976 | 3335	<0.001
Insurance			
Medicare	Ref	Ref	NI
Medicaid	1880	337 | 3423	0.017
Private	-1891	-2892 | -890	<0.001
No insurance	-692	-2551 | 1167	0.465
Hospital Bed Size			
Small	Ref	Ref	NI
Medium	8521	7216 | 9827	<0.001
Large	13046	11718 | 14375	<0.001
Teaching status			
Non-teaching	Ref	Ref	NI
Teaching	10803	9720 | 11885	<0.001

## Discussion

**Figure 2:**
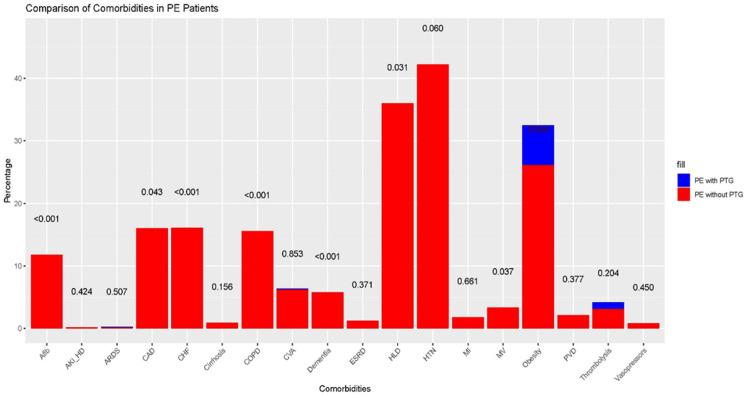
Bar chart PE: Pulmonary embolism; PTG: Prothrombin gene; p value <0.05: statistical significance; MI: myocardial infarction; ARDS: acute respiratory distress syndrome; HLD: hyperlipidemia; PVD: peripheral vascular disease; CHF: congestive heart failure; CAD: coronary artery disease; Afib: atrial fibrillation, HTN: hypertension; CVA: cerebrovascular accidents; COPD: chronic obstructive pulmonary disease; ESRD: end stage renal disease; AKI-HD: Acute kidney injury requiring hemodialysis; MV: mechanical ventilation

In our large, nationally representative study of patients admitted for PE, we found that individuals with the PGM G20210A mutation had notably lower in-hospital mortality rates than those without it. This was true even after considering various confounding factors like age, sex, comorbidities, and socioeconomic status. PGM carriers had an 87% reduced odds of in-hospital mortality. Patients with PGM presented with a higher prevalence of obesity and hyperlipidemia; they exhibited a lower prevalence of severe comorbid conditions, including atrial fibrillation, CHF, and COPD, which likely contributed to their improved survival (Figure 2). Although patients with the PGM mutation had a slightly longer average hospital stay of 0.52 days, this did not lead to significantly higher hospital charges [10]. 

Furthermore, those with PGM and pulmonary embolism demonstrated a more favorable clinical profile, showing lower mortality rates and fewer severe comorbidities compared to the control group. These results concur with earlier research, such as Cohen et al., which demonstrated that hospitalized PGM patients treated with standard therapy had lower rates of major bleeding and all-cause mortality [11]. Furthermore, our study confirms previous findings showing that PGM is more prevalent in white patients [12,13] with a lower prevalence among Black and Asian populations [14]. In line with other studies, our data also indicate that PGM patients are, on average, younger than those without the mutation [15], which likely contributes to better outcomes. Our results are consistent with data from the RIETE registry, an international database that similarly reported a younger age among PGM carriers [15].

One of the notable strengths of our study is its reliance on national estimates, which enhances the generalizability of our findings compared to earlier single-center or multicenter studies. Moreover, we have observed no significant differences in the prevalence of PGM between sexes, challenging previous reports that indicated a lower prevalence in females [16]. Our data, drawn from a larger and more diverse cohort, suggest no significant sex-based variation in PGM prevalence.

Our study offers several strengths. The use of the NIS dataset, which is nationally representative, allowed us to generate robust population-based estimates. Moreover, given the size of our sample, this study is one of the largest published to date on PGM in the United States. Additionally, because PGM was linked to hospitalization as a secondary diagnosis, we could identify whether patients were diagnosed with the mutation before, during or after their hospital stay [16].

However, there are several limitations to acknowledge. We could not differentiate between homozygous and heterozygous carriers of the PGM mutation, limiting our ability to fully stratify risk. Additionally, the NIS does not collect treatment data, meaning we could not assess which therapies patients received or the rationale behind clinical decisions [16,17]. As with any large dataset, missing data were a concern, which we addressed using multiple imputations. While we tried to adjust for confounding variables, the use of ICD-10 coding and our study's observational nature still leaves the possibility of residual or unknown confounding factors. Furthermore, without long-term follow-up data, our findings are limited to what occurred during the initial hospitalization, which might not fully reflect the longer-term outcomes for patients [17].

In closing, our national retrospective cohort study reveals an intriguing insight: patients with PE who have the PGM G20210A mutation seem to experience lower in-hospital mortality rates. This may be due to these patients generally being younger and having fewer severe health issues, which likely contributes to their better outcomes during hospitalization. Our results underscore the importance of considering genetic factors when assessing outcomes for patients with PE. These factors could provide valuable insights into their overall health and prognosis, helping us better understand the complexities of their conditions.

## Conclusions

This study demonstrates that PE patients carrying the prothrombin G20210A mutation experience significantly lower in-hospital mortality compared to non-carriers, despite a marginally longer hospital stay. This survival advantage may be attributed to the younger age and fewer severe comorbidities, such as atrial fibrillation and congestive heart failure, observed in PGM carriers. Interestingly, these patients showed higher rates of obesity and hyperlipidemia, highlighting the nuanced impact of genetic factors on clinical outcomes.

The findings suggest that genetic profiles like PGM should be considered in managing PE, as they may influence both prognosis and treatment approaches. While this study offers valuable insights, further research is necessary to explore long-term outcomes and potential differences in treatment strategies between homozygous and heterozygous PGM carriers. Understanding the role of genetic mutations, such as PGM, in PE can lead to more personalized and effective care, helping to improve survival and optimize healthcare resource use.
